# Xanthine oxidase inhibition attenuates insulin resistance and diet-induced steatohepatitis in mice

**DOI:** 10.1038/s41598-020-57784-3

**Published:** 2020-01-21

**Authors:** Tomoki Nishikawa, Naoto Nagata, Tetsuro Shimakami, Takashi Shirakura, Chieko Matsui, Yinhua Ni, Fen Zhuge, Liang Xu, Guanliang Chen, Mayumi Nagashimada, Taro Yamashita, Yoshio Sakai, Tatsuya Yamashita, Eishiro Mizukoshi, Masao Honda, Shuichi Kaneko, Tsuguhito Ota

**Affiliations:** 1 Department of Gastroenterology, Kanazawa University Graduate School of Medical Science, Kanazawa, Japan; 2 Department of Cell Metabolism and Nutrition, Advanced Preventive Medical Sciences Research Center, Kanazawa University Graduate School of Medical Science, Kanazawa, Japan; 3 Department of Cellular and Molecular Function Analysis, Kanazawa University Graduate School of Medical Science, Kanazawa, Japan; 4 Pharmaceutical Development Research Laboratories, Teijin Institute for Bio-Medical Research, Teijin Pharma Limited, Hino, Japan; 5 Division of Metabolism and Biosystemic Science, Department of Medicine, Asahikawa Medical University, Asahikawa, Japan

**Keywords:** Metabolic syndrome, Non-alcoholic fatty liver disease

## Abstract

Hyperuricemia drives the development of nonalcoholic fatty liver disease (NAFLD). Pharmacological inhibition of xanthine oxidase (XO), a rate-limiting enzyme for uric acid (UA) production, has been demonstrated to improve hepatic steatosis in diet-induced obese mice. However, it remains unclear whether inhibition of XO improves nonalcoholic steatohepatitis (NASH), a more advanced form of NAFLD, in terms of both liver inflammation and fibrosis. Here, we investigated the effects of febuxostat and allopurinol, two XO inhibitors clinically used for gout, on a mouse model of NASH. Furthermore, we conducted a single-arm, open-label intervention study with febuxostat for NAFLD patients with hyperuricemia. Despite a similar hypouricemic effect of the XO inhibitors on blood UA level, febuxostat, but not allopurinol, significantly decreased hepatic XO activity and UA levels in the NASH model mice. These reductions in hepatic XO activity and UA levels were accompanied by attenuation of insulin resistance, lipid peroxidation, and classically activated M1-like macrophage accumulation in the liver. Furthermore, in NAFLD patients with hyperuricemia, treatment with febuxostat for 24 weeks decreased the serum UA level, accompanied by reductions in the serum levels of liver enzymes, alanine aminotransferase and aspartate aminotransferase. XO may represent a promising therapeutic target in NAFLD/NASH, especially in patients with hyperuricemia.

## Introduction

Nonalcoholic fatty liver disease (NAFLD), one of the most common liver diseases worldwide^1^, is strongly associated with insulin resistance and features of metabolic syndrome such as obesity, hyperlipidemia, and type 2 diabetes^1,2^. NAFLD ranges from simple steatosis to more advanced nonalcoholic steatohepatitis (NASH) characterized by steatosis in combination with inflammation and fibrosis^3,4^. Besides insulin resistance, increased inflammatory cytokines, reactive oxygen species (ROS), and subsequent lipid peroxidation are thought to drive the progression of NASH, leading to liver cirrhosis and hepatocellular carcinoma^3,5^. Thus, in addition to conventional approaches based on of diet- and exercise-related adjunct therapies, an effective therapeutic approach is urgently needed for NASH.

Besides metabolic syndrome-related conditions, hyperuricemia, characterized by high serum uric acid (UA) levels, has also been linked to NAFLD. Several epidemiological studies have demonstrated that patients with NAFLD have significantly higher serum UA levels relative to controls, and elevated serum UA levels are an independent risk factor for NAFLD^6–8^. Notably, UA itself has been reported to promote *de novo* lipogenesis and induce insulin resistance, both *in vivo* and *in vitro*, through increased NADPH oxidase (NOX)-mediated ROS generation^9,10^ and activation of the NOD-like receptor family pyrin domain containing 3 (NLRP3) inflammasome^11^. These observations indicate that hyperuricemia plays a causative role in the development of NAFLD; it is not merely a consequence of this liver disease.

It is thought that hyperuricemia in NAFLD is primarily due to increased expression and/or activity of hepatic xanthine oxidase (XO)^12^. XO catalyzes the oxidation of hypoxanthine to xanthine, and then to UA, along with generation of ROS, such as superoxide anion and hydrogen peroxide^13–15^. Therefore, lowering UA by inhibiting XO could attenuate the liver damage seen in NAFLD by suppressing NLRP3 inflammasome activation and both NOX- and XO-mediated ROS production. In fact, a previous study demonstrated that the expression and activity of hepatic XO are significantly increased in high-fat diet-induced obese mice, while allopurinol, a classical XO inhibitor with a purine-like structure, improves insulin resistance and hepatic steatosis in these mice^11^. However, it remains unclear whether and how lowering UA by inhibiting XO improves NASH, which is characterized by more advanced liver inflammation and fibrosis. Thus, in the current study, we investigated the effects of pharmacological inhibition of XO with allopurinol or febuxostat, a nonpurine selective XO inhibitor, in mice fed a high-fat, -cholesterol, and -cholate diet (CL diet)^16,17^. Finally, we conducted a pilot intervention study with febuxostat for NAFLD patients with hyperuricemia.

## Results

### Febuxostat decreased hepatic XO activity and UA levels in mice fed a CL diet

**Figure 1:**
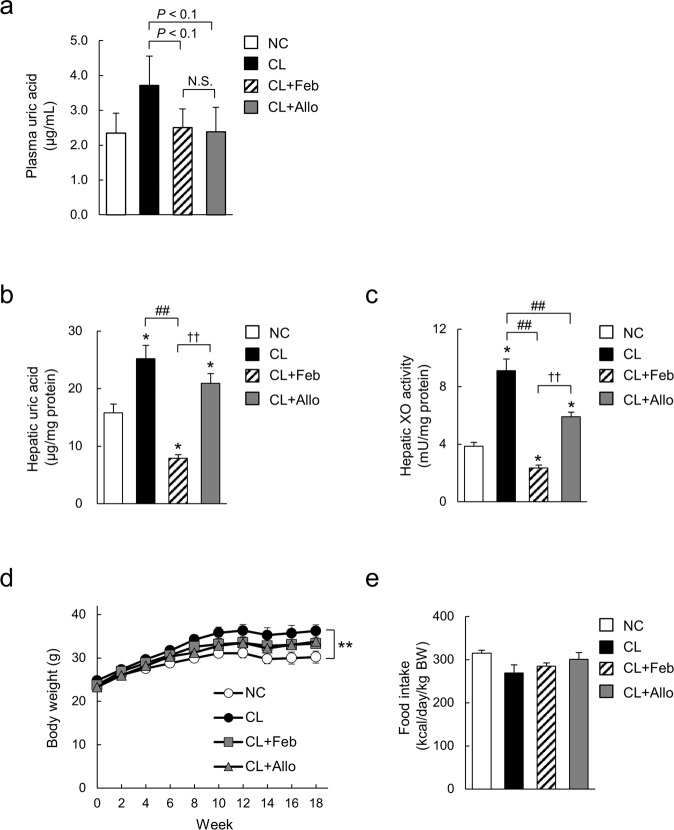
Febuxostat decreased hepatic levels of uric acid (UA) and xanthine oxidase (XO) activity in mice fed a high-fat, -cholesterol, and -cholate diet (CL) diet. (**a**) Plasma levels of UA in mice fed normal chow (NC), CL, CL diet containing 0.001% febuxostat (CL-Feb), or CL diet containing 0.003% allopurinol (CL-Allo) diet for 18 weeks. (**b**) Hepatic UA levels and (**c**) hepatic XO activity. (**d**) Body weight and (**e**) food intake at week 4. Data are mean ± SEM (n = 7–8/group). *P < 0.05, **P < 0.01 *vs*. NC; ^##^*P* < 0.01 *vs*. CL, ^††^*P* < 0.01 *vs*. CL + Feb.

To determine the effect of XO inhibitors on diet-induced NASH, C57BL/6J mice were split into four groups and fed NC, the CL diet, or the CL diet supplemented with either 0.001% (w/w) febuxostat (CL + Feb) or 0.003% (w/w) allopurinol (CL + Allo) for 18 weeks. Doses of febuxostat and allopurinol were selected based on a previous animal study showing a comparable hypouricemic effect in rodents^18^. First, we confirmed that administration of febuxostat and allopurinol conferred equivalent decreases in plasma UA levels of mice fed the CL diet (Fig. 1a). Next, we investigated hepatic UA levels and XO activity. Compared with control mice fed NC, mice fed the CL diet showed significantly elevated hepatic UA levels and XO activity (Fig. 1b,c). Febuxostat markedly decreased hepatic UA levels and XO activity in mice fed the CL diet (Fig. 1b,c). Allopurinol also significantly decreased hepatic XO activity (Fig. 1c); however, this decrease was not sufficient to significantly attenuate the elevation of hepatic UA levels caused by the CL diet (Fig. 1b). Mice fed the CL diet for 18 weeks exhibited significantly increased weight gain compared with the NC due to the higher fat content (Fig. 1d). CL + Feb and CL + Allo mice exhibited comparable body weight gain (Fig. 1d), fat weight (Table 1), and food intake (Fig. 1e) to the untreated CL mice. However, the increase in liver weight caused by the CL diet was alleviated by febuxostat, but not by allopurinol (Table 1).

**Table 1:** Metabolic parameters after 18 weeks of feeding.

	NC	CL	CL + Feb	CL + Allo
Epididymal fat (% of BW)	3.9 ± 0.3	5.0 ± 0.2*	4.5 ± 0.2	4.5 ± 0.2
Liver weight (% of BW)	4.1 ± 0.1	5.9 ± 0.3**	5.0 ± 0.2**^#^	5.6 ± 0.1**
Plasma TG (mg/dL)	142.6 ± 9.7	85.5 ± 5.1*	76.6 ± 6.5*	70.3 ± 4.7*
Plasma TC (mg/dL)	208.8 ± 27.4	382.1 ± 29.8*	380.0 ± 13.1*	415.6 ± 11.9*
Plasma FFAs (mmol/L)	1.51 ± 0.04	1.26 ± 0.05*	1.14 ± 0.04*	1.10 ± 0.07*
Plasma ALT (IU/L)	6.1 ± 1.9	95.5 ± 11.0**	29.3 ± 6.1^##^	29.2 ± 2.7^##^
Plasma AST (IU/L)	25.9 ± 5.2	105.3 ± 20.9**	62.6 ± 5.1^$^	73.2 ± 5.5*

Shown are epididymal fat pad weight, liver weight, plasma levels of triglycerides (TG), total cholesterol (TC), free fatty acids (FFAs), alanine aminotransferase (ALT), and aspartate aminotransferase (AST) in mice fed the indicated diets for 18 weeks. Plasma samples were collected from the mice fasted for 16 h. Measurements are reported as mean ± SEM (n = 7–8/group). *P < 0.05, **P < 0.01 vs. NC; ^$^*P* < 0.1, ^#^*P* < 0.05, ^##^*P* < 0.01 vs. CL. NC, normal chow; CL, high-fat, -cholesterol, and -cholate diet; CL + Feb, CL diet containing 0.001% febuxostat; CL + Allo, CL diet containing 0.003% allopurinol.

### Febuxostat improved glucose tolerance and insulin resistance

**Figure 2:**
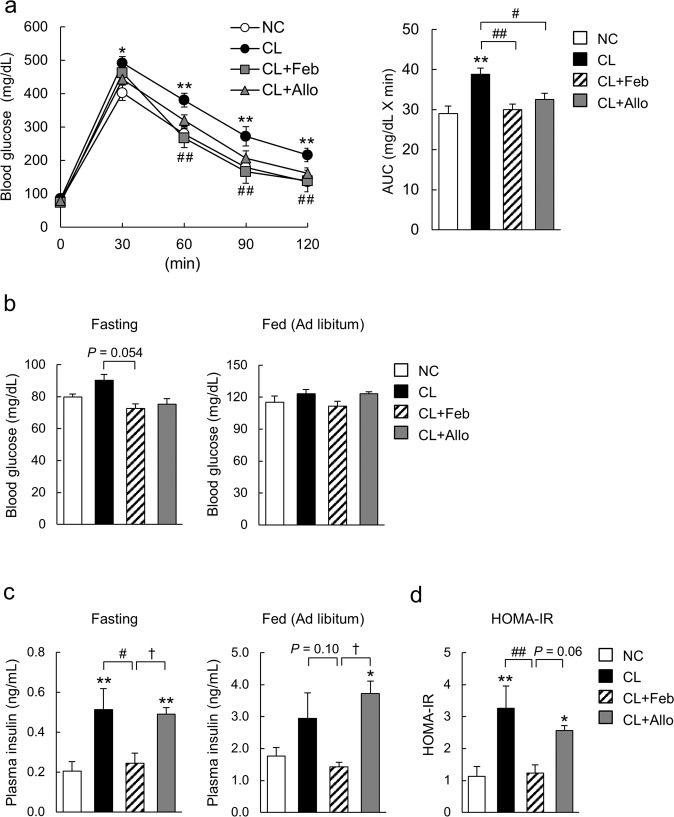
Febuxostat improved the glucose tolerance and insulin sensitivity of mice fed the CL diet. (**a**) Glucose tolerance test (GTT; 2 g/kg body weight) was performed after 12 weeks of feeding (n = 7–8 mice per group). Bar graphs represent area under the curve (AUC) calculations. (**b**) Blood glucose and (**c**) plasma insulin levels of mice fasted for 16 h or fed *ad libitum* (n = 6–7 mice per group). (**d**) Homeostatic model assessment of insulin resistance (HOMA-IR) was calculated from fasting plasma glucose and insulin concentrations (n = 6–7 mice per group). Data are presented as the mean ± SEM. *P < 0.05, **P < 0.01 vs. NC; ^#^*P* < 0.05, ^##^*P* < 0.01, vs^.^ CL; ^†^*P* < 0.05 *vs*. CL + Feb.

To determine whether XO inhibitors improve glucose metabolism, mice were subjected to a GTT. During the GTT, both febuxostat and allopurinol improved glucose tolerance in CL diet-fed mice (Fig. 2a). Compared with CL mice, CL + Feb mice showed a trend toward lower fasting blood glucose levels (Fig. 2b). Additionally, febuxostat significantly decreased plasma insulin concentrations in CL-fed mice under fasting conditions (Fig. 2c), resulting in lower homeostasis model assessment of insulin resistance (HOMA-IR), a marker for insulin resistance (Fig. 2d). In contrast, allopurinol did not affect fasting blood glucose levels, plasma insulin levels, or HOMA-IR relative to mice fed the CL diet (Fig. 2b–d).

### Febuxostat and allopurinol attenuated CL diet-induced lipid accumulation and fibrosis in the liver

**Figure 3:**
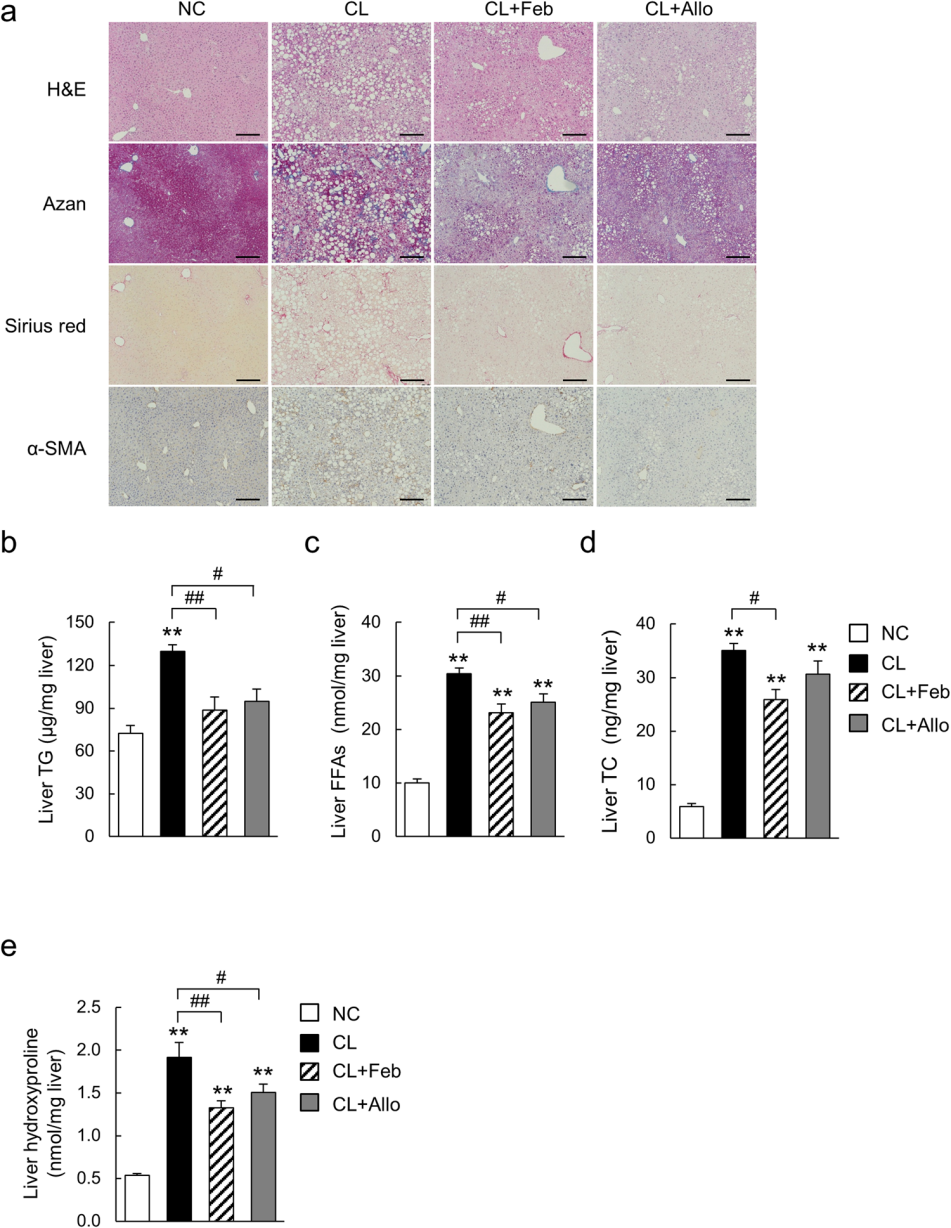
Febuxostat and allopurinol attenuated the development of hepatic steatosis and fibrosis in NASH mice. (**a**) Liver sections were stained with hematoxylin-eosin (H&E), Azan, and Sirius Red. α-smooth muscle actin (α-SMA)–positive hepatic stellate cells were detected by immunohistochemical staining. The original magnification was × 200. Scale bars represent 100 μm. (**b**) Triglycerides (TG), (**c**) free fatty acids (FFAs), and (**d**) total cholesterol (TC) levels in the livers. (**e**) Hydroxyproline content in the livers. Data are mean ± SEM (n = 7–8 mice per group). **P < 0.01 *vs*. NC; ^#^*P* < 0.05, ^##^*P* < 0.01 *vs*. CL. A.U., arbitrary unit.

H&E staining of liver sections from mice fed the CL diet for 18 weeks revealed clear evidence of micro- and macrosteatosis (Fig. 3a). In contrast, febuxostat and allopurinol attenuated both hepatic steatosis (Fig. 3a) and the increases in liver TG and FFAs levels associated with the CL diet (Fig. 3b,c), though plasma TG, TC, and FFAs levels were not decreased (Table 1). TC, a lipotoxic molecule involved in the development of experimental and human NASH, was significantly decreased in the livers of CL diet mice treated with febuxostat, but not allopurinol (Fig. 3d)^16,19^. Histological analyses with Sirius Red and Azan staining revealed that febuxostat and allopurinol attenuated the hepatic fibrosis induced by the CL diet (Fig. 3a). In parallel, the number of α-SMA-positive activated hepatic stellate cells, a major fibrogenic cell, increased in response to the CL diet, but was decreased in mice treated with febuxostat and allopurinol (Fig. 3a). Consistent with the histological findings, both febuxostat and allopurinol reduced hepatic hydroxyproline, a marker of collagen fiber content (Fig. 3e). Additionally, compared with the CL group, the lower levels of plasma ALT in the febuxostat- and allopurinol-treated groups indicated an attenuation of CL diet-induced liver damage (Table 1).

### Febuxostat alleviated CL diet-induced inflammation and oxidative stress in the liver

**Figure 4:**
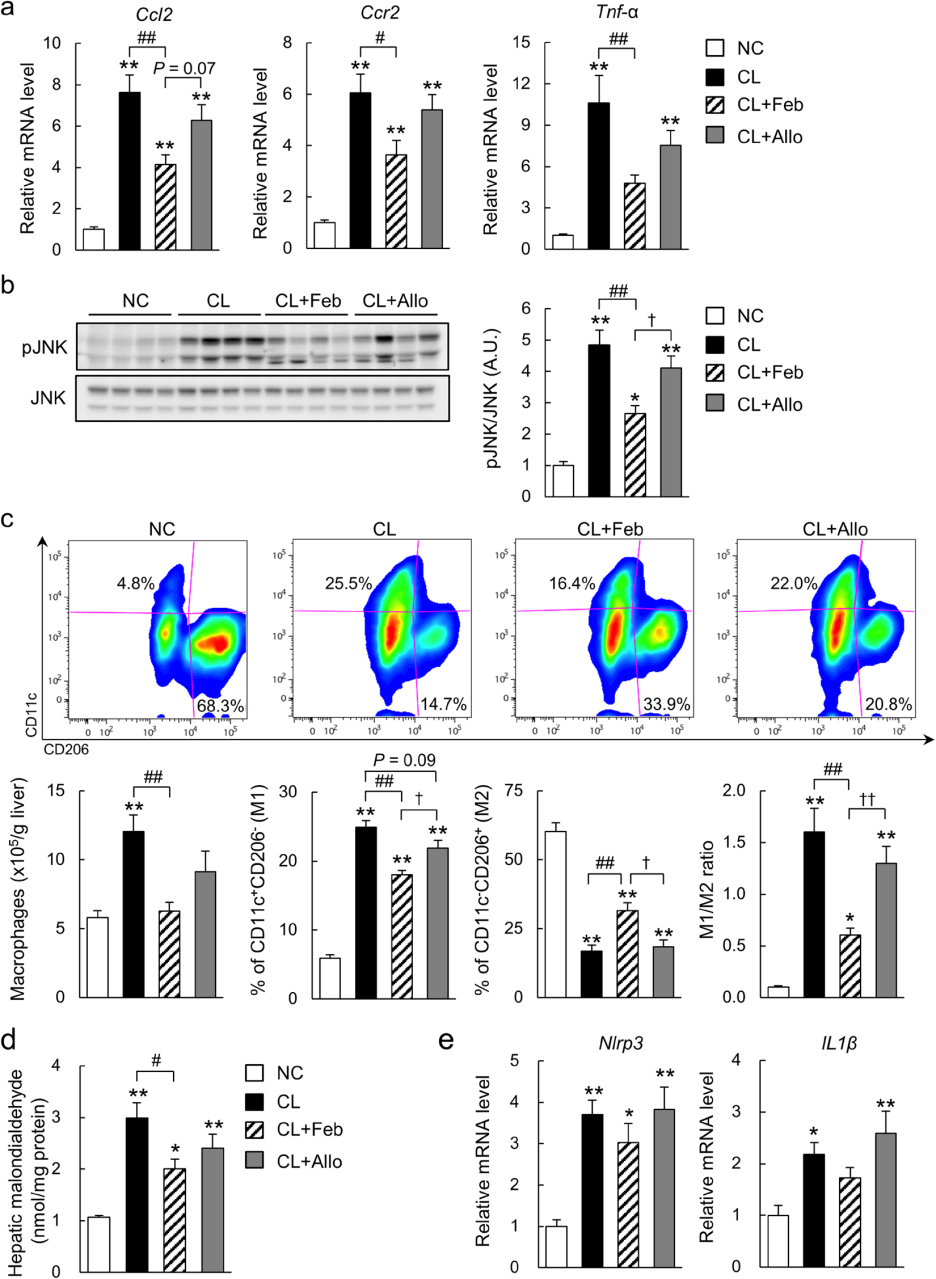
Febuxostat alleviated hepatic inflammation and lipid peroxidation in NASH mice. (**a**) Relative mRNA expression of Ccl2, Ccr2, and tumor necrosis factor-α in the liver of mice fed the indicated diet for 18 weeks (n = 7–8 mice per group). (**b**) Representative immunoblot of phosphorylated c-Jun N-terminal kinase (p-JNK) (Thr^183^/Tyr^185^) and JNK using liver lysates. Each lane represents a liver lysate from a different animal (n = 8 mice per group). Bar graphs represent normalized p-JNK/JNK data from two independent experiments. (**c**) FACS analysis of liver macrophages of mice fed the indicated diet (n = 6–8 mice per group). Macrophages are defined as propidium iodide^−^CD45^+^NK1.1^−^CD3^−^CD19^−^TER119^−^CD11b^+^F4/80^+^ cells. M1-like and M2-like macrophages are defined as CD11c^+^CD206^−^ and CD11c^−^CD206^+^, respectively. Bar graph shows the number of liver macrophages, the percentage of M1- and M2-like macrophages, and the M1/M2 ratio. (**d**) Hepatic levels of malondialdehyde (n = 7–8 mice per group). (**e**) Relative mRNA expression of NOD-like receptor family pyrin domain containing 3 (Nlrp3) and interleukin-1β (Il-1β) in the liver of mice fed the indicated diet for 18 weeks (n = 7–8 mice per group). Data are presented as mean ± SEM. *P < 0.05, **P < 0.01 vs. NC; ^#^*P* < 0.05, ^##^*P* < 0.01 vs^.^ CL; ^†^*P* < 0.05, ^††^*P* < 0.01 *vs*. CL + Feb. A.U.: arbitrary unit.

Chemokine (C-C motif) ligand 2 (Ccl2) increases recruitment of Ccl2 receptor (Ccr2)-positive inflammatory monocytes into the liver^20^. In response to the CL diet, the resulting influx of Ccr2-positive cells and liver-resident macrophages (Kupffer cells) produces a large amount of proinflammatory mediators and promotes insulin resistance and NASH in mice^21,22^. Here, we observed a marked induction of *Ccl2*, *Ccr2*, and tumor necrosis factor-α (*Tnf-a*) in the liver of CL mice, which was significantly attenuated in CL + Feb mice but not CL + Allo mice (Fig. 4a). In parallel, febuxostat significantly suppressed CL diet-induced c-Jun N-terminal kinase (JNK) activation (Fig. 4b). Tissue macrophages are phenotypically heterogeneous and are characterized according to their polarization/activation state as either proinflammatory M1-like macrophages or anti-inflammatory M2-like macrophages^23^. To determine the number of macrophages (identified as propidium iodine^−^NK1.1^−^CD3^−^CD19^−^TER119^−^CD45^+^CD11b^+^F4/80^+^ cells) in the liver and their polarization state as M1-like (CD11c^+^CD206^−^) or M2-like (CD11c^-^CD206^+^), we analyzed hepatic immune cells with FACS. As previously reported^21^, the total number of hepatic macrophages increased 2-fold in mice fed the CL diet compared with the NC (Fig. 4c). CL + Feb, but not CL + Allo, mice exhibited a decrease in hepatic macrophage content compared with CL mice. Of note, compared with CL mice, CL + Feb mice exhibited a 28% decrease in the number of M1-like macrophages, whereas the proportion of M2-like macrophages increased by 15%, resulting in a predominantly M2-like macrophage population (Fig. 4c). In contrast, allopurinol led to a decrease in the proportion of M1-like macrophages but did not alter the proportion of M2-like macrophages or the M1/M2 ratio (Fig. 4c).

XO mediates ROS production in inflammatory disease^15,24^. In line with increased XO activity in the liver of CL mice (Fig. 1d), hepatic levels of malondialdehyde, a marker of lipid peroxidation, were increased by the CL diet (Fig. 4d). Febuxostat, but not allopurinol, significantly attenuated lipid peroxidation in the liver (Fig. 4d). In addition, we found that mice fed the CL diet exhibited significant up-regulation of hepatic mRNA expression of *Nlrp3* and interleukin-1β (*Il-1β*), indicating inflammasome activation in the liver (Fig. 4e). However, neither febuxostat nor allopurinol suppressed mRNA expression of *Nlrp3* or *Il-1β* in the liver of mice fed the CL diet (Fig. 4e).

### Febuxostat alleviated NAFLD in patients with hyperuricemia

**Figure 5:**
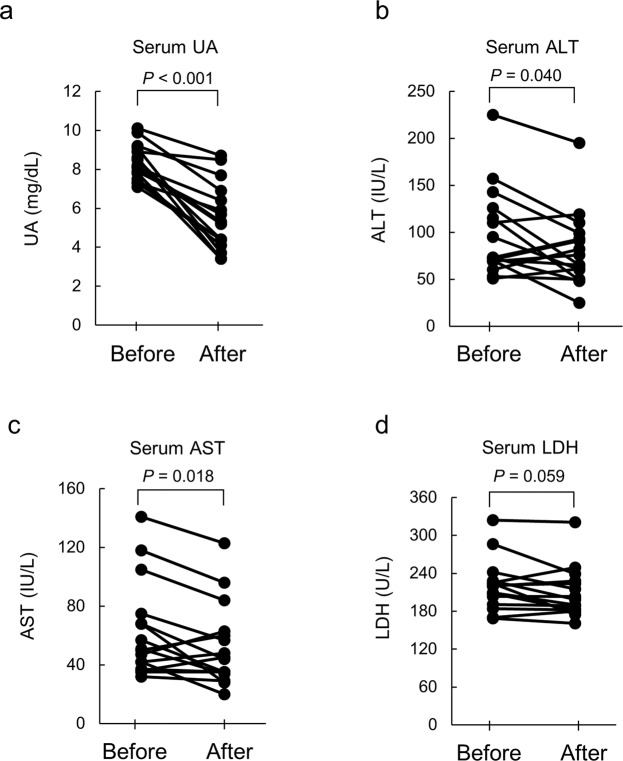
Febuxostat alleviated NAFLD in human subjects with hyperuricemia. Changes in (**a**) serum levels of UA, (**b**) alanine aminotransferase (ALT), (**c**) aspartate aminotransferase (AST), and (**d**) lactate dehydrogenase (LDH) of participants before and after 24 weeks of intervention with febuxostat. Each line between the circle symbols represents a change in serum levels for a given parameter. Sixteen out of twenty-five participants with moderate liver injury (serum ALT levels > 50 IU/L at baseline) were selected and visualized. *P* values were analyzed by paired samples t-test.

Since our animal study revealed the beneficial effects of XO inhibition on NAFLD, we next extended our study to human NAFLD patients. We conducted a pilot intervention study using febuxostat against NAFLD in patients with hyperuricemia to determine whether febuxostat reduces serum levels of ALT and AST, two markers of liver injury. Twenty-five patients met the eligibility criteria and consented to participate in the study. All participants completed the study (Supplemental Table 3). Although a significant decrease in serum UA levels and a trend toward decreased serum LDH levels were observed after 24 weeks of febuxostat treatment, other parameters, including serum ALT, AST, ALP, and γ-GTP were not significantly changed (Supplemental Table 4). However, in 16 of 25 patients with moderate liver injury (ALT > 50 IU/L) before treatment, febuxostat effectively reduced serum UA levels [median (interquartile range), before: 8.2 (7.7–9.0); after: 5.3 (4.3–6.5) mg/dL, *P* < 0.001] accompanied by a significant decrease in serum levels of ALT [before: 73.0 (69.8–117.8); after: 70.5 (57.5–94.5) IU/L, *P* = 0.040] and AST [before: 50.5 (40.8–69.8); after: 44.5 (34.8–60.8) IU/L, *P* = 0.018], and with a trend toward decreased serum LDH levels [before: 220 (200–226); after: 199 (182–226) U/L, *P* = 0.059] (Fig. 5). Additionally, using liver biopsies obtained from a NAFLD patient (patient No. 1 in Supplemental Table 3), we confirmed that hepatic steatosis was improved by the treatment with febuxostat [(steatosis grade) before: 3; after: 2], although inflammation and ballooning were not improved (Supplemental Fig. 1). Together, these findings imply that febuxostat may have therapeutic potential for NAFLD in patients with hyperuricemia.

## Discussion

In the current study, we demonstrated that both febuxostat and allopurinol alleviated glucose intolerance, hepatic steatosis and fibrosis, in mice fed the CL diet. Despite a similar hypouricemic effect of the XO inhibitors on blood, febuxostat, but not allopurinol, significantly decreased hepatic UA levels and XO activity in NASH model mice. This reduction in hepatic UA levels and XO activity was accompanied by more effective prevention of certain features of NASH, including insulin resistance, lipid peroxidation, classically activated M1-like macrophage accumulation, and liver inflammation. Finally, we demonstrated that febuxostat has the potential to improve NAFLD in patients with hyperuricemia.

The CL diet was shown to induced glucose intolerance, insulin resistance, hepatic lipid peroxidation, and steatohepatitis in mice, as previously reported^16,21,22^. These metabolic abnormalities were associated with elevated hepatic UA levels and XO activity. Here, we show that both febuxostat and allopurinol alleviated glucose intolerance, hepatic steatosis, and fibrosis in mice fed the CL diet, without affecting food intake, body mass, or fat pad weight. Our results suggest that the effect of XO inhibitors was not associated with decreased food intake, body mass, or adiposity. We demonstrated that febuxostat more potently lowered hepatic UA levels and XO activity in mice fed the CL diet relative to allopurinol. Additionally, CL + Feb mice exhibited decreased HOMA-IR, hepatic lipid peroxidation, JNK activation, and a lower ratio of M1/M2 liver macrophages compared to CL + Allo mice. Several previous studies support the notion that differences in hepatic oxidative stress levels may account for the difference in efficacy between febuxostat and allopurinol. First, oxidative stress-mediated JNK activation induces lipid accumulation through the inhibition of insulin signaling^25–27^. Second, an increase in lipid peroxides causes inflammation and fibrosis via activating liver macrophages and hepatic stellate cells^28,29^. Third, the antioxidant carotenoids, astaxanthin and β-cryptoxanthin, not only decrease CL diet-induced lipid peroxidation, but also alleviate steatohepatitis, including hepatic steatosis, inflammation, and fibrosis^21,22^. However, we do not exclude the possibility that XO inhibitors mitigated CL diet-induced steatohepatitis through mechanisms independent of oxidative stress.

Recently, Nakatsu *et al*. demonstrated that febuxostat suppressed hepatic steatosis, oxidative stress, inflammatory cytokine expression, and fibrosis, but not insulin resistance, in another NASH model: mice fed a high-fat diet containing trans fatty acids^30^. Here, despite a similar degree of hypouricemic effects seen in response to XO inhibitors, febuxostat but not allopurinol significantly lowered hepatic UA levels in NASH mice. This reduction in hepatic UA levels in CL + Feb mice was accompanied by more effective prevention of certain features of NASH, such as insulin resistance, hepatic lipid peroxidation, and inflammatory activation of liver macrophages. Thus, our findings extend those of the previous study that demonstrated the efficacy of febuxostat in NASH mice, by revealing the pathophysiological impact of hepatic UA levels and XO activity rather than plasma UA levels on the development of NAFLD. This finding deserves further investigation in studies directly comparing XO inhibitors with plasma UA-lowering agents, such as a urate transporter 1 (URAT1) inhibitor, on NASH development. Of note, in the clinical study done by George *et al*., allopurinol, but not the URAT1 inhibitor probenecid, improved endothelial function in chronic heart failure, despite similar lowering of plasma UA levels^31^. That study also showed that the mechanism of action of allopurinol, with respect to its improvement of endothelial function, lies in its ability to abolish XO-mediated vascular oxidative stress rather than reducing UA.

Febuxostat reduced the infiltration and activation of liver macrophages, resulting in the attenuation of hepatic inflammation in CL diet-fed mice. Hepatic inflammation mediated by macrophage/monocyte-derived proinflammatory cytokines promotes lipogenesis by inducing insulin resistance^32^. In fact, the depletion of liver macrophages by liposomal clodronate ameliorates hepatic steatosis and insulin sensitivity in mice fed a high-fat diet^33^. Furthermore, specific ablation of M1-like macrophages restores insulin sensitivity in diet-induced obese mice^34^. Additionally, M2-like liver macrophages have been reported to mitigate NAFLD by promoting apoptosis of M1-like macrophages^35^. Therefore, the decreased number of hepatic macrophages and their M2-dominant polarization account, at least in part, for the attenuation of insulin resistance and steatohepatitis in febuxostat-treated CL diet-fed mice, even though the mRNA expression of Nlrp3 or Il-1β in the liver is not decreased. However, we do not exclude the possibility that the effects of XO inhibitors on CL diet-induced NAFLD are mediated by NLRP3 inflammasome-dependent mechanisms, since the efficacy and mechanism of action of XO inhibitors against NAFLD might be dependent on the dietary model. This point warrants further investigations.

Finally, our clinical study demonstrated that febuxostat has the potential to reduce liver damage in NAFLD. Febuxostat, with its non-purine structure, selectively inhibits both the oxidized and reduced form of XO^36,37^. In contrast, allopurinol and its oxidized active metabolite, oxypurinol, inhibit only the oxidized form of XO, but also inhibit enzymes involved in purine and pyrimidine metabolism, since they are hydroxypyrazolopyrimidine analogues of hypoxanthine and xanthine. These structure-related mechanisms are thought to underlie the more favorable toxicity profile, and more potent and longer-acting hypouricemic effect, of febuxostat relative to allopurinol^38,39^. Taken together, the animal and clinical studies presented here demonstrated that UA-lowering therapy with febuxostat could be effective against NAFLD/NASH associated with hyperuricemia. However, we do not exclude the possibility that allopurinol or other XO inhibitors can prevent the progression of NAFLD in humans. Further studies, including large, long-term randomized clinical trials are therefore warranted.

## Methods

### Animals and diets

Seven-week-old male C57BL/6J mice were purchased from Charles River Laboratories (Yokohama, Japan). After 1 week of adaptation, the mice were divided into four groups and fed for 18 weeks as follows: (1) normal chow [NC; 10% kcal from fat, CRF-1; Charles River, Wilmington, MA (n = 8)], (2) CL diet [CL; 60% of calories from fat, containing 1.25% cholesterol and 0.5% sodium cholate, #D06061403; Research Diets Inc., New Brunswick, NJ (n = 8)], (3) CL diet containing 0.001% febuxostat [CL + Feb; Teijin Pharma Ltd., Tokyo, Japan (n = 8)], or (4) CL diet containing 0.003% allopurinol [CL + Allo; Merck KGaA, Darmstadt, Germany (n = 8)]. The CL diets containing febuxostat and allopurinol were both prepared by Research Diets. All mice were maintained on a 12/12-h light/dark cycle and given free access to food and water. The animal experiment was repeated twice and the results of one of the two independent experiments are presented. All animal procedures were performed in accordance with the Guidelines for the Care and Use of Laboratory Animals of Kanazawa University, and all animal protocols were approved by the institutional animal care and use committee of Kanazawa University.

### XO activity measurement

Plasma and hepatic XO activity were measured using the pterin-based assay^40^. Briefly, frozen liver tissues were homogenized with potassium phosphate buffer, pH 7.4, containing 1 mM EDTA and protease inhibitors. The homogenates were centrifuged at 12,000 g for 15 min at 4 °C, and supernatants were collected. Either supernatant or plasma was co-incubated with 50 μM pterin solution to assay XO activity. After 60 min incubation at 37 °C, fluorometric assays were performed to calculate the production of isoxanthopterin. Activity of purified XO derived from buttermilk (Merck KGaA) and liver tissue protein concentration were measured and used to normalize the sample activity.

### UA measurement

UA levels in plasma and liver were measured as described previously, with minor modifications^41^. Plasma samples were supplemented with 100 μM allopurinol to suppress xanthine oxidoreductase activity. Frozen liver tissues were homogenized with potassium phosphate buffer, containing 100 μM allopurinol and 1 mM potassium oxonate, to avoid synthesis and degradation of UA, and centrifuged at 20,000 g for 5 min at 4 °C. Plasma and liver homogenate was added to a mixture of water and methanol. The resulting solution was mixed thoroughly on a vortex mixer for 5 s and centrifuged for 5 min at 20,000 g at 4 °C. The supernatants were subjected to UA measurement. Plasma and liver tissue concentrations of UA were measured by liquid chromatography coupled with tandem mass spectrometry (LC-MS/MS) on a Prominence UFLC system (Shimadzu, Kyoto, Japan) using an ODP2 HP-2D column (Shodex, Tokyo, Japan). The column oven was maintained at 40 °C, the flow rate was 0.25 mL/min, and the injection volume was 2 μL (plasma) or 1 μL (liver tissue). Separation was performed using a quaternary mobile phase consisting of water/ammonium acetate (1,000:1, v/v) (50%) and methanol (50%), with a flow rate of 0.25 mL/min. Electrospray ionization tandem mass spectrometry (ESI-MS/MS) analyses were performed on an API 5000 instrument (AB Sciex, Framingham, MA) equipped with a TurboIonSpray® source operated under multiple reaction monitoring and negative ion mode. For an internal standard solution, 5-ethyluracil solution diluted with water was used. Calibration curves for the peak area ratio of the analyte to the internal standard against the analyte concentration were obtained by weighted (1/×2, × = concentration) linear regression. The concentrations of the analyte in unknown samples were obtained from the regression curve.

### Metabolic measurements

Hepatic lipids were extracted with chloroform/methanol (2:1), as described previously^42^, and the extract was assayed using an enzymatic colorimetric method (Wako Pure Chemical, Osaka, Japan). Plasma triglycerides (TG), total cholesterol (TC), free fatty acids (FFAs), aspartate aminotransferase (AST), alanine aminotransferase (ALT), and insulin levels were measured as described previously^22^. Liver malondialdehyde was measured using a colorimetric assay kit (Cayman Chemicals, Ann Arbor, MI). For food intake measurements, the mice were individually housed in a metabolic cage for 7 days during week 4. A glucose tolerance test (GTT) was conducted after a 16-h fast, following 12 weeks of feeding. After baseline blood collection, mice were injected intraperitoneally with 2 g/kg glucose. Blood glucose values were measured using a glucometer (Sanwa Kagaku Kenkyusyo, Nagoya, Japan), before and at 30, 60, 90, and 120 min following injection. To assess liver collagen content, hydroxyproline levels were measured using a spectrophotometric assay, as reported previously^16^.

### Histological examination and immunohistochemistry

Paraffin-wax-embedded liver sections were stained with hematoxylin-eosin (H&E), Azan, and Sirius Red, as described previously^16^. For immunohistochemical analysis of α-smooth muscle actin (α-SMA), sections were immunostained with monoclonal mouse anti-human α-SMA (Agilent, Santa Clara, CA). This was followed by application of the immunoperoxidase technique using an Envision kit (Agilent). Peroxidase activity was identified by reaction with 3′,3′-diaminobenzidine (Sigma-Aldrich, St. Louis, MO).

### Quantitative real-time PCR

Total RNA was isolated from frozen liver samples using a GenElute Mammalian Total RNA Miniprep Kit (Sigma-Aldrich). cDNA was synthesized using a High-Capacity cDNA Reverse Transcription Kit (Applied Biosystems, Carlsbad, CA). Quantitative real-time PCR was then performed on a CFX384 machine (Bio-Rad, Hercules, CA) using SYBR Green Master Mix (Applied Biosystems), as described previously^21^. The primers used for quantitative real-time PCR are shown in Supplementary Table 1.

### Immunoblotting

Liver tissues were homogenized in RIPA lysis buffer (Millipore, Billerica, MA) supplemented with protease and phosphatase inhibitors (Sigma-Aldrich). Proteins were resolved by SDS-PAGE and transferred to polyvinylidene difluoride membranes (Bio-Rad). Immunoblot of lysates was performed with primary antibodies (Supplemental Table 2); after incubation with appropriate secondary antibodies (Cell Signaling Technology, Danvers, MA), proteins were visualized with a chemiluminescent substrate (Millipore) and imaged using a camera system (LAS-4000 mini; GE Healthcare, Little Chalfont, UK). Pixel intensities of immunoreactive bands were quantified using Image Studio Lite software (ver. 5.2; LI-COR Biosciences, Lincoln, NE).

### Fluorescence-activated cell sorting (FACS) analysis

The left lobes of the livers were gently lysed and digested for 20 min at 37 °C using type IV collagenase (Sigma-Aldrich) and type I deoxyribonuclease (Sigma-Aldrich) in phosphate-buffered saline containing 2% bovine serum albumin (pH 7.4). Non-parenchymal cells were incubated with Fc Block (BD Biosciences, San Jose, CA) and then with fluorochrome-conjugated antibodies (Supplemental Table 2). Cells were analyzed using a FACSAria II instrument (BD Bioscience), as described previously^43^. Hepatic macrophages were identified as propidium iodine^−^NK1.1^−^CD3^−^CD19^−^TER119^−^CD45^+^CD11b^+^F4/80^+^ cells. Data analysis was performed using FlowJo software (Tree Star, Ashland, OR).

### Protocol, patient eligibility criteria, and outcome of the clinical study

We conducted a single-arm, multicenter, open-label intervention study from August 2012 through July 2018 in Japan. The study was approved by the Ethics Committee of Kanazawa University Hospital. The study has been registered with UMIN-CTR (#UMIN000008686). The study was conducted in accordance with the International Ethical Guidelines and Declaration of Helsinki. Participants were recruited from among a cohort of Japanese outpatients. Inclusion criteria were age >20 years, serum ALT ≥ 31 IU/L, serum UA ≥ 7.0 mg/dL, and diagnosis of NAFLD using ultrasonography based on hepatorenal contrast or hepatic morphological change. Patients were excluded if they had serious liver diseases; were suspected of acute liver failure, viral hepatitis, or other serious diseases including cardiac disease, renal dysfunction, and poorly controlled diabetes mellitus; habitually consumed alcohol (more than 30 g/day for males and 20 g/day for females); or took any of the following medicines, which can affect serum UA levels, within 4 weeks before entering this study: allopurinol, benzbromarone, probenecid, bucolome, febuxostat, losartan, fenofibrate, loop diuretic, or thiazide diuretic. Written informed consent was obtained from all participants. Patients received 10 mg of febuxostat once daily as the starting dose, which was then increased to 20 mg/day every 4 weeks, up to 40–60 mg/day for a total 24 of weeks. The primary outcome was the difference in serum ALT levels between before and after receiving febuxostat for 24 weeks. The secondary outcomes were blood biochemical parameters, including UA, AST, γ-glutamyl transpeptidase (γ-GTP), lactate dehydrogenase (LDH), and alkali phosphatase (ALP). Liver biopsies were obtained from one patient who provided informed consent, before and at the end of the study. Biopsy specimens were scored by a single hepatopathologist using the NASH Clinical Research Network Histologic Scoring System^44^.

### Statistical analysis

Animal study data are expressed as mean ± SEM. *P* < 0.05 was considered statistically significant. Statistical differences between pairs of groups were determined by a two-tailed Student’s *t*-test. An overall difference among more than two groups was determined by one-way ANOVA; if significant, differences between pairs of groups were determined using a Tukey *post hoc* test. In the human clinical study, statistical differences before versus after treatment for each individual were determined by a paired samples *t*-test. All calculations were performed using SPSS software (ver. 24.0; IBM Corp., Armonk, NY).

## Supplementary information

Supplementary Information.

## Data Availability

No datasets were generated or analyzed during the current study.
