# Supplementary material for: Xanthine oxidase inhibition attenuates insulin resistance and diet-induced steatohepatitis in mice
Source: Sci Rep. 2020 Jan 21;10:815. doi: 10.1038/s41598-020-57784-3 (PMC6972756; doi:10.1038/s41598-020-57784-3)

Xanthine oxidase inhibition attenuates insulin resistance and diet-induced steatohepatitis in mice

Tomoki Nishikawa[1,a], Naoto Nagata[2,3,a], Tetsuro Shimakami[1], Takashi Shirakura[4], Chieko Matsui[4], Yinhua Ni[2], Fen Zhuge[2], Liang Xu[2], Guanliang Chen[2], Mayumi Nagashimada[2], Taro Yamashita[1], Yoshio Sakai[1], Tatsuya Yamashita[1,2], Eishiro Mizukoshi[1], Masao Honda[1], Shuichi Kaneko[1], Tsuguhito Ota[2,5,*]

[1] Department of Gastroenterology, Kanazawa University Graduate School of Medical Science, Kanazawa, Japan

[2] Department of Cell Metabolism and Nutrition, Advanced Preventive Medical Sciences Research Center, Kanazawa University Graduate School of Medical Science, Kanazawa, Japan

[3] Department of Cellular and Molecular Function Analysis, Kanazawa University Graduate School of Medical Science, Kanazawa, Japan

[4] Pharmaceutical Development Research Laboratories, Teijin Institute for Bio-Medical Research, Teijin Pharma Limited, Hino, Japan

[5] Division of Metabolism and Biosystemic Science, Department of Medicine, Asahikawa Medical University, Asahikawa, Japan

[a] These two authors contributed equally to this work.

Supplementary Table 1. Primers used for quantitative RT-PCR.

**Gene/Primer**	**Forward**	**Reverse**
Ccl2	AGGTCCCTGTCATGCTTCTGG	CTGCTGCTGGTGATCCTCTTG
Ccr2	ATTCTCCACACCCTGTTTCG	GATTCCTGGAAGGTGGTCAA
IL1β	CTGTGACTCGTGGGATGATG	GGGATTTTGTCGTTGCTTGT
Nlrp3	AGCCTTCCAGGATCCTCTTC	CTTGGGCAGCAGTTTCTTTC
Tnfa	CCCACACCGTCAGCCGATTT	GTCTAAGTACTTGGGCAGATTGACC

Supplementary Table 2. Antibodies used for immunoblotting, immunohistochemistry, and FACS analysis.

Antibodies	Supplier
pT^{183}/Y^{185}JNK	Cell Signaling Technology #9255
JNK	Cell Signaling Technology #9258
α-smooth muscle actin, clone 1A4	Agilent #M0851
PerCP-Cyanine5.5-conjugated NK1.1	eBioscience #45-5941
PerCP-Cyanine5.5-conjugated CD3	eBioscience #45-036
PerCP-Cyanine5.5-conjugated CD19	eBioscience #45-0193
PerCP-Cyanine5.5-conjugated TER-119	eBioscience #45-5921
Allophycocyanin-eFluor 780-conjugated CD45	eBioscience #47-0451
Phycoerythrin-conjugated CD11c	eBioscience #12-0114
eFluor 450-conjugated Ly-6G (Gr-1)	eBioscience #48-5931
PE/Cy7-conjugated F4/80	Biolegend #123113
Alexa Fluor 647-conjugated CD206	Biolegend #141712
PE-Texas Red-conjugated CD11b	Invitrogen #RM2817

Supplemental Table 3. Laboratory data of individual patient before and after the treatment with febuxostat for 24 weeks.

			ALT (IU/L)		AST (IU/L)		ALP (U/L)		LDH (U/L)		γ-GTP (U/L)		UA (mg/dL)	
No.	Age	Sex	Before	After	Before	After	Before	After	Before	After	Before	After	Before	After
1	65	F	143	99	118	96	375	381	209	190	327	360	8.2	5.3
2	72	M	30	18	35	23	283	321	186	179	24	23	7.1	5.1
3	44	M	225	195	141	123	171	171	221	224	108	100	7.1	4.4
4	28	M	157	110	75	57	247	269	170	180	120	93	7.9	5.7
5	43	M	71	65	37	35	240	231	324	321	30	36	8.1	6.4
6	41	M	73	93	47	63	312	292	225	249	247	251	9.1	3.7
7	41	M	46	35	30	25	288	258	173	177	142	140	8.2	4.8
8	36	M	126	65	57	35	270	212	220	N/A	155	130	10.1	8.7
9	31	M	110	119	50	60	262	201	203	203	66	95	9.2	7.7
10	65	M	73	48	68	44	250	239	286	239	31	33	7.8	3.4
11	49	M	71	91	42	48	186	203	220	228	32	47	9.9	6.9
12	60	M	37	46	50	75	102	138	148	166	105	320	7.3	4.8
13	53	F	70	25	42	20	406	288	228	199	289	65	8.5	4.4
14	46	F	45	68	45	55	359	418	354	265	38	46	7.3	2.6
15	37	M	34	60	19	29	354	251	133	142	350	127	10.4	7.6
16	53	M	51	61	32	29	367	374	208	182	25	26	8.1	5.2
17	51	M	42	58	37	43	155	168	245	230	44	54	10.6	6.5
18	49	F	53	50	105	84	409	445	169	161	621	673	8.6	3.4
19	48	M	115	49	68	28	188	200	224	175	263	169	8.9	8.5
20	45	M	30	50	24	42	145	169	199	211	53	63	7.2	6.8
21	33	M	95	60	51	34	240	296	242	215	124	100	7.5	4.1
22	47	M	40	34	25	24	180	170	161	163	30	30	8.4	9.5
23	30	M	47	80	18	32	268	265	203	197	23	35	7.6	7.0
24	27	M	69	76	35	35	146	140	191	189	51	96	7.3	5.9
25	50	M	60	82	36	45	128	106	184	182	57	71	7.3	4.4

M, male; F, female; N/A, not available; ALT, alanine aminotransferase; ALP, alkali phosphatase; AST, aspartate aminotransferase; LDH, lactate dehydrogenase; γ-GTP, γ-glutamyl transpeptidase;

UA, uric acid.

Supplemental Table 4. Patient characteristics before and after the treatment with febuxostat for 24 weeks.

Characteristics	Median (interquartile range)		
	Before	After	*P*-value
**ALT, IU/L (n = 25)**	69 (45-95)	61 (49-82)	0.242
**AST, IU/L (n = 25)**	42 (35-57)	42 (29-57)	0.227
**ALP, U/L (n = 25)**	250 (180-312)	239 (171-292)	0.571
**LDH, U/L (Before: n = 25; After: n = 24)**	208 (184-225)	194 (179-225)	0.064
**γ-GTP, U/L (n = 25)**	66 (32-155)	93 (46-130)	0.680
**Uric acid, mg/dL (n = 25)**	8.1 (7.3-8.9)	5.3 (4.4-6.9)	< 0.001

ALT, alanine aminotransferase; ALP, alkali phosphatase; AST, aspartate aminotransferase; LDH, lactate dehydrogenase; γ-GTP, γ-glutamyl transpeptidase; UA, uric acid. Statistical differences between before and after treatment in same individual were determined by a paired samples t-test.

Supplemental Figure 1. Liver histology in a NAFLD patient with hyperuricemia before and after the treatment with febuxostat. Representative H&E-stained liver sections from one subject with NAFLD before and after treatment with febuxostat for 24 weeks. Scale bars = 400 μm

Supplemental Figure 1

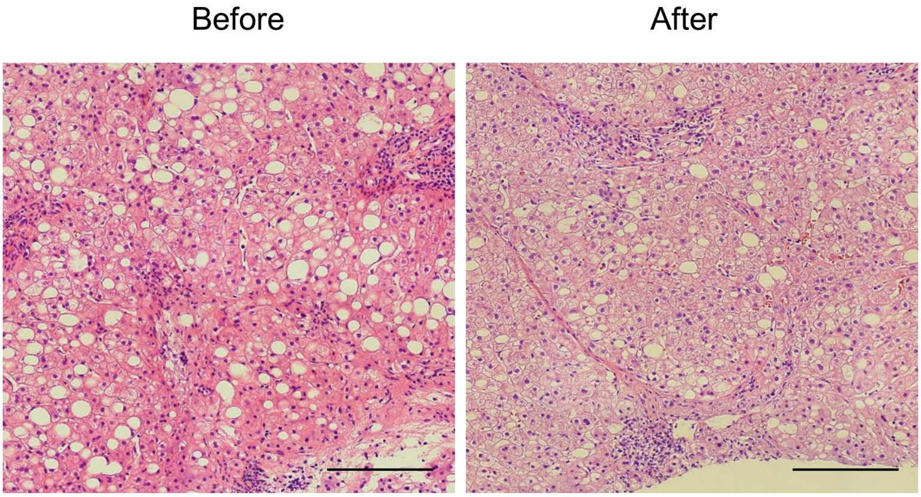

Uncropped blots
Figure 4b

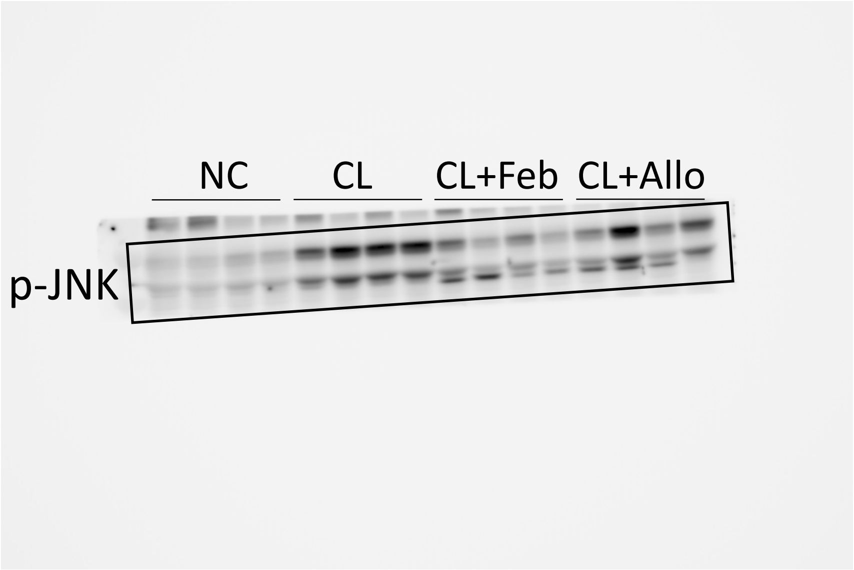

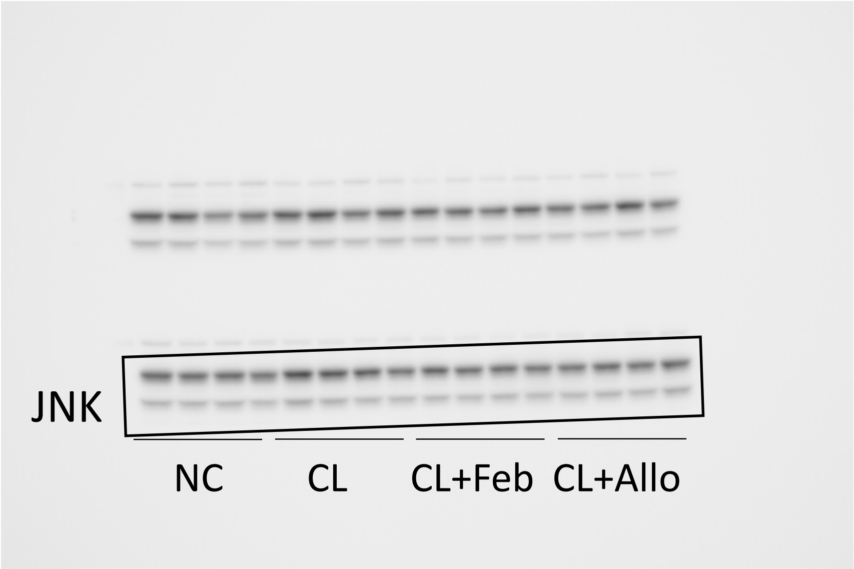

Supplement: Supplementary file 1 — Supplementary Information. [file 41598_2020_57784_MOESM1_ESM.pdf]
